# Prognostic Value of Dynamic Segmented Neutrophil to Monocyte (SeMo) Ratio Changes in Patients with Moderate to Severe Traumatic Brain Injury

**DOI:** 10.3390/diagnostics14161836

**Published:** 2024-08-22

**Authors:** Lin Chang, Yu-Jun Lin, Ching-Hua Tsai, Cheng-Shyuan Rau, Shiun-Yuan Hsu, Ching-Hua Hsieh

**Affiliations:** 1 Department of Neurosurgery, Kaohsiung Chang Gung Memorial Hospital, Chang Gung University College of Medicine, Kaohsiung 83301, Taiwan; d24706@cgmh.org.tw (L.C.); lyr1022@cgmh.org.tw (Y.-J.L.); ersh2127@cgmh.org.tw (C.-S.R.); 2 Department of Trauma Surgery, Kaohsiung Chang Gung Memorial Hospital, Chang Gung University College of Medicine, Kaohsiung 83301, Taiwan; tsai1737@cloud.cgmh.org.tw (C.-H.T.); ah.lucy@hotmail.com (S.-Y.H.); 3 Department of Plastic Surgery, Kaohsiung Chang Gung Memorial Hospital, Chang Gung University College of Medicine, Kaohsiung 83301, Taiwan

**Keywords:** Glasgow Coma Scale (GCS), monocytes, mortality, neutrophils, traumatic brain injuries (TBI)

## Abstract

Background: Traumatic brain injury (TBI) is a leading cause of morbidity and mortality in trauma patients, necessitating reliable prognostic tools. The segmented neutrophil-to-monocyte (SeMo) ratio, indicative of the inflammatory response, has emerged as a valuable biomarker. This study evaluates the prognostic value of dynamic changes in the SeMo ratio in predicting outcomes for patients with moderate to severe TBI. Methods: A retrospective analysis was conducted on data from 1118 TBI patients admitted to the surgical intensive care unit at a level I trauma center between January 2009 and December 2020. Patients were selected based on an Abbreviated Injury Scale (AIS) score ≥ 3 in the head region. Initial and follow-up SeMo ratios were calculated upon admission and 48–72 h later, respectively. The dynamic SeMo ratio was defined as the difference between the second and initial SeMo ratios. Statistical analyses included receiver operating characteristic (ROC) curve analysis to determine the optimal threshold for mortality prediction, and comparative analysis of clinical outcomes. Results: The study cohort included 121 deceased and 997 surviving patients. Deceased patients had significantly higher second SeMo ratios (20.9 ± 16.1 vs. 15.8 ± 17.2, *p* = 0.001) and dynamic SeMo ratios (2.4 ± 19.8 vs. −2.1 ± 19.5, *p* = 0.019) than those survival patients. In the multivariate analysis, the dynamic SeMo is a significant independent risk factor for in-hospital mortality (OR 1.01, 95%CI: 1.01–1.03, *p* = 0.031). The optimal cut-off for the dynamic SeMo ratio was 5.96, above which patients exhibited higher mortality (21.4% vs. 8.5%, *p* < 0.001), higher adjusted mortality (adjusted odds ratio: 2.98; 95% confidence interval: 1.95–4.56; *p* = 0.005), and longer hospital stays (23.6 days vs. 19.7 days, *p* = 0.005). Discussion: Dynamic SeMo ratio changes serve as a prognostic marker for in-hospital mortality and hospital stay duration in moderate to severe TBI patients. A higher dynamic SeMo ratio indicates increased risk, highlighting the importance of early monitoring and intervention. Future prospective studies should validate these findings and explore integration with other biomarkers for enhanced prognostication.

## 1. Introduction

Traumatic brain injury (TBI) is a primary cause of morbidity and mortality among trauma victims. With a high frequency of long-term neurological consequences, TBI frequently necessitated immediate attention and more medical resources for therapy. As a result, a quick and easy tool for predicting the prognosis and monitoring the patient’s condition is beneficial to patient care. Trauma injury, including TBI, would activate the innate immune system, engaging microglia and astrocytes, resulting in a prolonged inflammatory response defined by the release of cytokines and chemokines [1]. Circulating neutrophils, lymphocytes, monocytes, and platelet counts may reflect the patient’s immunological status as well as the intensity of the inflammatory response. Furthermore, various blood cells component ratios, such as the neutrophil-to-lymphocyte ratio (NLR), platelet-to-lymphocyte ratio (PLR), and lymphocyte-to-monocyte ratio (LMR), have been proposed as valuable biomarkers for reflecting inflammation or immune status in trauma patients [2,3] and those with TBI [4,5]. These blood cell ratios are not only cost-efficient and easily obtainable, but they also provide important insights into systemic immune responses, allowing physicians to stratify risk and customize therapies more effectively for trauma patients [2,3,4,6].

In clinical practice, the segmented neutrophil-to-monocyte (SeMo) ratio has emerged as another biomarker for the ratio of blood cell components that can indicate an individual’s bodily state of inflammation [7,8,9,10,11]. An elevation in segmented neutrophils is associated with excessive expression of inflammatory cytokines, which is connected to the occurrence of numerous system organ failures and mortality [7]. In contrast, immune paralysis, which is defined by monocyte deactivation, has been contributing to poor patient outcomes [12]. The SeMo ratio has been associated with immune dysfunction scores, cytokine expression, and monocyte activity, providing information on the patient’s immunological response and prognosis [3]. SeMo ratios greater than 15 have been identified to predict the occurrence of moderate-to-severe stroke in patients with acute ischemic stroke [13]. Defort P, et al. revealed that the SeMo ratio is an independent predictor of in-hospital mortality in severe TBI patients [5]. Researchers also found that the SeMo ratio outperformed LMR and NLR, with a magnitude comparable to the conventional indicators of C-reactive protein and erythrocyte sedimentation rate [14].

Studies have shown that an increase in SeMo ratio measurement can identify critical sepsis patients admitted to the intensive care unit (ICU) and predict survival outcomes [15]. Researchers have reported that using the dynamic SeMo ratio, which is defined as a shift in the SeMo ratio within a short time after admission, along with the leukocyte count, is a quick and straightforward method for risk stratification [15]. This method also reveals how the immune system and inflammatory cytokines are controlled in these sepsis patients [15]. Under the hypothesis that the changes in dynamic SeMo ratio might aid in predicting patient outcomes, the aim of this study was to evaluate the capacity to predict in-hospital mortality and LOS in hospitals of dynamic SeMo ratio changes in patients with moderate to severe TBI, using a retrospective review of the registered trauma database at a level I trauma center.

## 2. Materials and Methods

### 2.1. Study Design

This study was designed as a retrospective observational cohort study. Data were collected from patients who were admitted to the surgical ICU with moderate to severe TBI over a specified period. The study aimed to evaluate the prognostic significance of the dynamic segmented neutrophil to monocyte (SeMo) ratio in predicting in-hospital mortality and LOS in hospitals. The dynamic SeMo ratio was calculated as the difference between the SeMo ratio measured at admission and a second measurement taken 48 to 72 h later. Statistical analyses were conducted to determine the optimal SeMo ratio cut-off for mortality prediction and to assess its association with clinical outcomes.

### 2.2. Setting

The study was conducted at Kaohsiung Chang Gung Memorial Hospital, a level I trauma center located in Kaohsiung, Taiwan. The hospital is equipped with a surgical ICU that manages a large number of trauma patients, providing an ideal setting for studying the outcomes of patients with moderate to severe TBI. The data were sourced from the hospital’s Trauma Registry System, which includes detailed records of trauma patients admitted between January 2009 and December 2020.

### 2.3. Participants and Study Size

**Figure 1:**
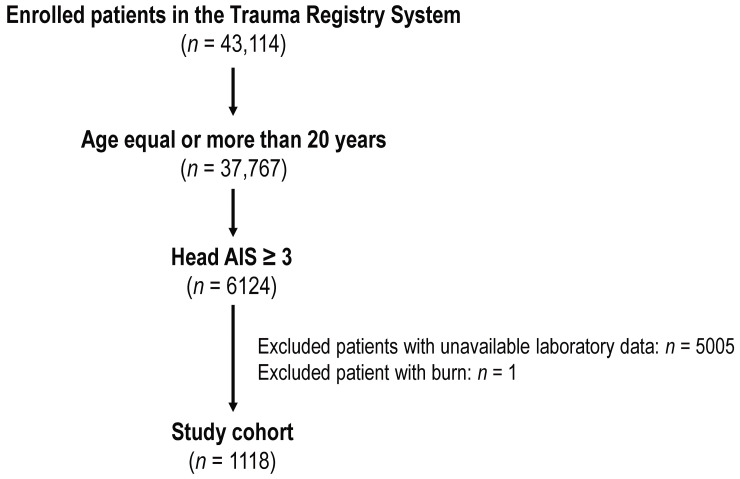
The study cohort selected from the trauma database of enrolled participants.

Figure 1 illustrates the process of selecting the study cohort from the initial sample of 43,414 trauma patients in the study period. We selected patients admitted to the surgical ICU with an AIS score of 3 or higher in the head area, indicating moderate to severe TBI. Patients with unavailable laboratory data (*n* = 5005) and burn injuries (*n* = 1) were excluded, leaving a final cohort of 1118 patients, including 121 deaths and 997 survivors.

### 2.4. Variables

The collected variables included demographics like age and sex as well as medical history, current conditions, blood counts (neutrophils and monocytes), SeMo ratio, Abbreviated Injury Scale (AIS), Glasgow Coma Scale (GCS), Injury Severity Score (ISS), length of stay (LOS) and mortality in the hospital. The AIS is a globally recognized injury scoring system developed to classify and describe the severity of injuries. It assigns a numerical score from 1 (minor) to 6 (unsurvivable) to different types of injuries, based on their severity. Each injury is categorized by body region, including the head, face, chest, abdomen, extremities, and external. The AIS helps predict patient outcomes by correlating the severity of injuries with the likelihood of mortality or morbidity. The first version of the scale was published in 1969 [16] with the latest updates in 2015 [17]. The GCS is a clinical tool used to assess a patient’s level of consciousness following a traumatic brain injury based on three aspects of responsiveness: eye opening, verbal response, and motor response [18]. Each component is scored separately and then combined to provide an overall score ranging from 3 to 15, with higher scores indicating better neurological function. Eye opening is scored from 1 to 4, verbal response from 1 to 5, and motor response from 1 to 6. The GCS is widely used in emergency and intensive care settings to quickly assess the severity of brain injuries, guide treatment decisions, and predict patient outcomes. The ISS is a trauma scoring system used to assess the overall severity of injuries in patients with multiple traumas. To calculate the ISS, the body is divided into six regions: head/neck, face, chest, abdomen/pelvis, extremities, and external. The three most severe injuries, each from a different body region, are selected. The AIS scores of these three injuries are then squared and summed to produce the ISS. The resulting score ranges from 1 to 75, with higher scores indicating more severe trauma and a greater likelihood of mortality. An ISS of 75 is assigned to any patient with at least one AIS score of 6 (unsurvivable injury), regardless of the severity of other injuries [19]. LOS was defined as the days from admission to discharge or in-hospital death of the patients.

### 2.5. Data Sources

We collected data from the Trauma Registry System and medical records from the study population. We collected laboratory data on the neutrophil and monocyte counts in uL, along with the derived SeMo ratio, from patients when they first arrived at the emergency room (known as the initial SeMo ratio) and 48 to 72 h later (known as the second SeMo ratio). The calculation of the dynamic SeMo ratio involved subtracting the initial SeMo ratio from the second SeMo ratio. The outcome of this study was the in-hospital mortality rate; the second outcome was hospital LOS.

### 2.6. Statistical Methods

The categorical data was compared using either the two-sided Fisher exact or Pearson chi-square tests, giving the computation of the odds ratio (OR) and 95% confidence interval (CI). Levene’s test was employed to evaluate the homogeneity of variances in variables that are continuous. The analysis of normally distributed continuous data was conducted using an unpaired Student’s *t*-test, and the results are reported as the mean value plus or minus the standard deviation. The Mann–Whitney U-test was used to evaluate non-normally distributed data, such as GCS and ISS. The results are reported as the median and interquartile range (IQR, Q1–Q3). The optimal threshold for predicting death was determined by calculating the area under the curve (AUC) of the receiver operating characteristic (ROC) curve using the Youden index, which maximizes both sensitivity and specificity. Afterwards, the patients were categorized based on the optimal threshold of the dynamic SeMo ratio in order to assess their mortality risk. Subsequently, adjusted odds ratios (AOR) were calculated for variables that had a substantial contribution to the patient’s mortality. Univariate and multivariate analyses identified independent risk factors for in-hospital mortality. A *p*-value of less than 0.05 was set as the statistical significance level. All statistical analyses in this study were carried out using IBM SPSS Statistics for Windows (version 23.0; IBM Corp., Armonk, NY, USA).

## 3. Results

Table 1 compares clinical and demographic characteristics between deceased and surviving patients with moderate to severe TBI. The analysis includes 121 deceased patients and 997 survivors. The deceased patients were older on average (63.3 ± 20.3 years vs. 57.9 ± 19.2 years, *p* = 0.004) and had higher second SeMo ratios (20.9 ± 16.1 vs. 15.8 ± 17.2, *p* = 0.001). However, there was no difference in initial SeMo ratios between deceased and surviving patients. Deceased patients also exhibited a significant increase in dynamic SeMo ratio (2.4 ± 19.8 vs. −2.1 ± 19.5, *p* = 0.019). Notably, the deceased patients had an average substantial increase in dynamic SeMo ratio while the survival patients had decreased dynamic SeMo ratio. Among comorbidities, coronary artery disease (CAD), cerebral vascular accident (CVA), and end-stage renal disease (ESRD) were more prevalent in the deceased group. Deceased patients had significantly lower GCS scores (median 8 vs. 14, *p* < 0.001), with a higher proportion in severe coma (GCS 3–8: 54.5% vs. 28.1%, *p* < 0.001). The ISS was also higher in deceased patients (median 25 vs. 20, *p* < 0.001), with a greater proportion having an ISS of ≥25 (67.8% vs. 33.6%, *p* < 0.001). Finally, the length of hospital stay was shorter for deceased patients (15.7 ± 14.3 days vs. 21.0 ± 16.5 days, *p* = 0.001), reflecting the critical condition leading to earlier in-hospital mortality.

**Figure 2:**
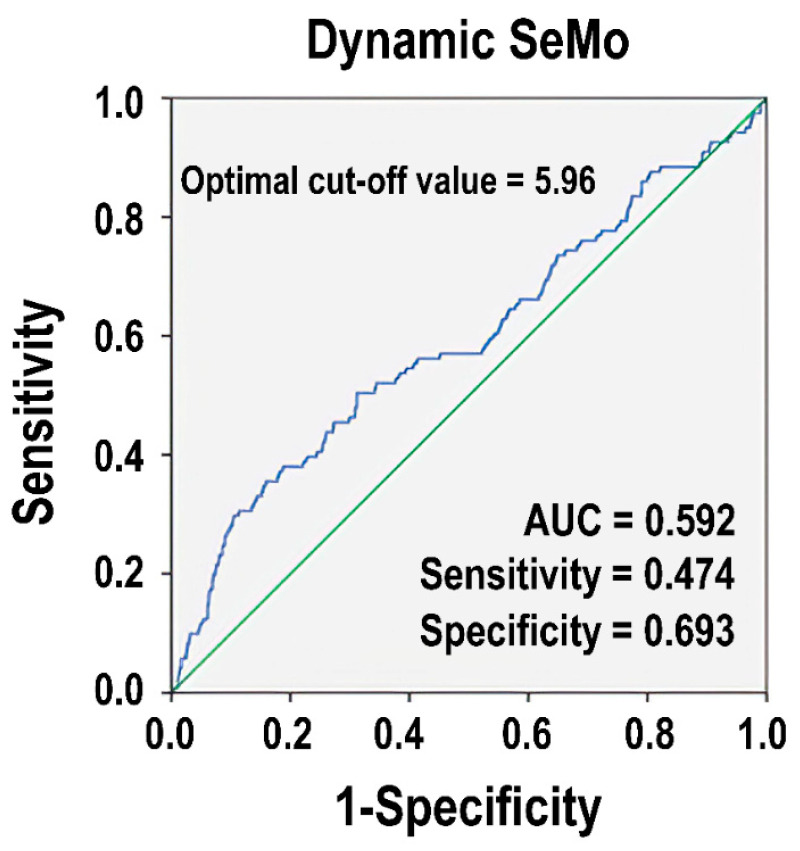
Illustration of the receiver operating characteristic (ROC) curve analysis for the dynamic segmented neutrophil to monocyte (SeMo) ratio (blue line) in predicting in-hospital mortality in patients with moderate to severe traumatic brain injury. The green line indicates an AUC = 0.5.

Figure 2 depicts an ROC curve for the dynamic SeMo ratio in predicting mortality. The curve demonstrates the trade-off between sensitivity and specificity, indicating the predictive value of the dynamic SeMo ratio as a diagnostic tool for predicting in-hospital mortality of patients in the study cohort. An optimal cut-off point for the dynamic SeMo ratio was 5.96, which achieved the highest sensitivity (47.4%) and specificity (69.3%) for predicting patient outcomes (AUC = 0.592, 95%CI: 0.522–0.632, *p* = 0.003). 

We performed a comparative analysis of patient groups divided by the optimal dynamic SeMo ratio (Table 2), with one group having a ratio greater than or equal to 5.96 (*n* = 201) and the other group having a ratio less than 5.96 (*n* = 917). The findings reveal no significant difference in sex distribution, age, or comorbidities between groups. It was found that people in the group with a high dynamic SeMo ratio had lower GCS scores (median: 12 vs. 13, *p* = 0.014), more severe comas (38.3% vs. 29.3%, *p* = 0.013), more deaths (21.4% vs. 8.5%, *p* < 0.001), and longer hospital stays (23.6 ± 18.2 days vs. 19.7 ± 15.9 days, *p* = 0.005) than people in the low dynamic SeMo ratio group. There was no significant difference between these two groups regarding ISS (median ISS: 20 [16–27] vs. 20 [16–25], *p* = 0.216). When age, CAD, CVA, ESRD, GCS, and ISS are taken into account, patients with a high dynamic SeMo ratio have almost three times higher death rates than patients with a low dynamic SeMo ratio (AOR 2.98; 95%CI 1.95–4.56; *p* = 0.005).

As shown in Table 3, the dynamic SeMo ratio went up significantly in the patients who died, while it went down significantly in the patients who survived. This is why we want to compare the groups of patients whose dynamic SeMo ratio was greater than or equal to 0 (*n* = 412) to those whose ratio was less than 0 (*n* = 706). There are no significant differences in sex distribution, age, or most comorbidities except ESRD and ISS between the two groups, according to the findings. A patient with a dynamic SeMo ratio greater than 0 had a significantly lower GCS score (12 [7–15] vs. 13 [8–15], *p* = 0.024), a higher rate of severe coma (35.0% vs. 28.6%, *p* = 0.027), a higher rate of death (15.3% vs. 8.2%, *p* < 0.001), and a longer hospital stay (22.1 ± 17.2 days vs. 19.5 ± 15.8 days, *p* = 0.011). Taking into account age, CAD, CVA, ESRD, GCS, and ISS, patients with an increased dynamic SeMo ratio have nearly three times higher death rates than those with a decreased dynamic SeMo ratio (AOR 2.01; 95%CI 1.30–3.01; *p* = 0.011).

Table 4 presents the results of the univariate and multivariate analyses identifying factors associated with mortality in adult trauma patients. The univariate analysis revealed that older age, pre-existing CVA, CAD, and ESRD, a lower GCS, a higher ISS, and a higher dynamic SeMo were significantly associated with increased mortality. In the multivariate analysis, a dynamic SeMo (OR 1.01, 95%CI: 1.01–1.03, *p* = 0.031) is a significant independent risk factor for in-hospital mortality. Additionally, older age (OR 1.03, *p* < 0.001), the presence of ESRD (OR 5.11, *p* < 0.001), lower GCS scores (OR 0.87, *p* < 0.001), and higher ISS (OR 1.06, *p* < 0.001) remained significantly associated with increased mortality.

## 4. Discussion

The study found that patients with moderate to severe TBI who had a high dynamic SeMo ratio had a greater likelihood of death and a longer duration of hospitalization, indicating a greater need for medical intervention. In contrast, for deceased patients, the length of hospital stay was shorter than those survival patients (15.7 ± 14.3 days vs. 21.0 ± 16.5 days, *p* = 0.001), reflecting the critical condition leading to earlier in-hospital mortality. In the multivariate analysis, a dynamic SeMo is a significant independent risk factor for in-hospital mortality. The prognostic model, which predicts the probability of in-hospital mortality, indicates that an optimal cut-off value of 5.96 for the dynamic SeMo ratio would identify patients at high risk for mortality, in contrast to the reported normal dynamic SeMo of less than 7 [15], a value which is derived from the study on consecutive sepsis patients in medical ICU, a condition different from this study on the trauma patients with moderate to severe TBI. Furthermore, in the aforementioned study, the term “dynamic SeMo” was defined as the difference between the SeMo ratio on day 3 and the SeMo ratio on day 1 [15]. However, in the present study, the dynamic SeMo was calculated using the SeMo ratio at 2–3 days after the initial measurement. The selection is made based on a practical consideration for ICU care, as not all patients would have blood laboratory testing on the third day following admission. This may also contribute to the variation in the identified dynamic SeMo value. As noted in this study, the dynamic SeMo ratio went up significantly in the patients who died, while it went down significantly in the patients who survived. An increased SeMo ratio 2–3 days after the injury may indicate a poor inflammatory or immunological response in the patients, which is connected with a worse result. Tsai CH et al. found similar results when they presented a delta-SeMo ratio, which is a ratio of the second SeMo ratio measured 2–3 days after admission divided by the initial SeMo ratio. Trauma ICU patients with a delta-SeMo ratio ≥ 1.038 had nearly double the adjusted mortality risk compared to those with a ratio < 1.038 [6]. 

The acquisition of both neutrophils and monocytes is easy and accurate during medical care. Monitor neutrophils and monocytes alone have long been used as diagnostic tools to follow the progression trend of the patients. The dynamic SeMo ratio, acting as an indicator of immune inflammation and reflecting a person’s current physiological condition, can serve as a rapid diagnostic or monitoring tool for predicting potential progression trends in patients with moderate to severe TBI. Implementing the dynamic SeMo ratio as a biomarker in clinical settings can significantly enhance the early risk stratification and management of patients with moderate to severe TBI. Studies have shown that integration of SeMo and other inflammatory biomarkers can be useful in a number of clinical settings, especially when used to predict outcomes in critical care [20]. Measuring the change in SeMo ratio upon ICU admission and on the third day can help identify patients at higher risk of poor outcomes, help guide more intensive monitoring and tailored therapeutic interventions. Integrating dynamic SeMo ratio data with other established biomarkers carries the potential to enhance prognostication accuracy. 

However, this study measured the second SeMo ratio 2–3 days later. The intriguing change in the SeMo ratio over time in the ICU warrants further investigation to elucidate its significance in clinical care. Furthermore, there are some other limitations to this study. First, the retrospective design and data from a single tertiary trauma center may introduce selection bias and limit generalizability to other populations. The study focused solely on in-hospital mortality, excluding patients who died upon arrival or after discharge, potentially underestimating the true mortality rate. We did not assess long-term outcomes or post-discharge complications. The analysis excluded important confounding factors like specific treatments and interventions (e.g., surgeries, blood transfusions, medications) that could influence the inflammatory response and SeMo ratio detected at different times. Neutrophil and monocyte counts, influenced by infections, stress, and medications, influence the dynamic SeMo ratio. The lack of control over these potential confounders impacted the accuracy of the dynamic SeMo ratio as a prognostic marker. Notably, in this study, the population was made up of patients with moderate to severe TBI, so if only one of these populations had been taken, such as with severe TBI, other results could have been obtained. At last, the second measurement time is critical, as a longer duration would most likely be meaningless, whilst a shorter time would be less sensitive to change. If the expression levels of each component could be detected every day after admission to the ICU, the calculated ratio might be more useful and accurate in reflecting the clinical status. However, this needed to be validated by more investigation. While the study provides valuable insights, its limitations underscore the need for prospective, multicenter studies with comprehensive data collection and control for confounding factors to validate these findings and enhance their generalizability.

## 5. Conclusions

This study demonstrates that dynamic changes in the segmented neutrophil-to-monocyte (SeMo) ratio are significant predictors of in-hospital mortality and hospital stay duration in patients with moderate to severe traumatic brain injury (TBI). A higher dynamic SeMo ratio correlates with increased risk and poorer outcomes. Implementing this biomarker in clinical settings may enhance early risk stratification and guide more effective management strategies. Future prospective studies are essential to validate these findings and improve their generalizability across diverse patient populations.

## Figures and Tables

**Table 1:** Comparison between patients who were deceased and survival.

Variables	Death *n* = 121	Survival *n* = 997	OR (95%CI)	*p*
Sex				0.195
Male, *n* (%)	85 (70.2)	641 (64.3)	1.31 (0.87–1.98)	
Female, *n* (%)	36 (29.8)	356 (35.7)	0.76 (0.51–1.15)	
Age	63.3 ± 20.3	57.9 ± 19.2	-	0.004
SeMo (Initial)	18.5 ± 11.1	17.9 ± 10.2	-	0.553
Neutrophil (count/uL)	8989.5 ± 5157.6	9619.6 ± 5044.4	-	0.196
Monocyte (count/uL)	565.9 ± 359.1	612.1 ± 384.4	-	0.209
SeMo (Second)	20.9 ± 16.1	15.8 ± 17.2	-	0.001
Neutrophil (count/uL)	8662.5 ± 4096.9	7905.8 ± 3513.1	-	0.054
Monocyte (count/uL)	566.5 ± 389.9	612.2 ± 319.6	-	0.217
Dynamic SeMo ratio	2.4 ± 19.8	−2.1 ± 19.5	-	0.019
Comorbidities				
DM, *n* (%)	24 (19.8)	209 (21.0)	0.93 (0.58–1.50)	0.773
HTN, *n* (%)	51 (42.1)	374 (37.5)	1.21 (0.83–1.78)	0.321
CAD, *n* (%)	17 (4.0)	73 (7.3)	2.07 (1.18–3.64)	0.010
CHF, *n* (%)	2 (1.7)	9 (0.9)	1.85 (0.39–8.64)	0.430
CVA, *n* (%)	14 (11.6)	49 (4.9)	2.53 (1.35–4.74)	0.003
ESRD, *n* (%)	11 (9.1)	23 (2.3)	4.24 (2.01–8.92)	<0.001
GCS, median (IQR)	8 (3–14)	14 (8–15)	-	<0.001
3–8, *n* (%)	66 (54.5)	280 (28.1)	3.07 (2.09–4.51)	<0.001
9–12, *n* (%)	19 (15.7)	151 (15.1)	1.04 (0.62–1.75)	0.872
13–15, *n* (%)	36 (29.8)	566 (56.8)	0.32 (0.21–0.49)	<0.001
ISS, median (IQR)	25 (16–29)	20 (16–25)	-	<0.001
1–15, *n* (%)	5 (4.1)	117 (11.7)	0.32 (0.13–0.81)	0.011
16–24, *n* (%)	34 (28.1)	545 (54.7)	0.32 (0.21–0.49)	<0.001
≥25, *n* (%)	82 (67.8)	335 (33.6)	4.16 (2.78–6.22)	<0.001
LOS in hospital (days)	15.7 ± 14.3	21.0 ± 16.5	-	0.001

CAD = coronary artery disease; CHF = congestive heart failure; CI = confidence interval; CVA = cerebral vascular accident; DM = diabetes mellitus; ESRD = end-stage renal disease; GCS = Glasgow Coma Scale; HTN = hypertension; IQR = interquartile range; ISS = injury severity score; LOS = length of stay; OR = odds ratio, SeMo = Neutrophil to Monocyte ratio.

**Table 2:** Comparison of patients with dynamic SeMo greater than or equal to 5.96 to those with a ratio less than 5.96, an optimal cut-off value identified from the receiver operating characteristic (ROC) curve analysis.

Variables	Dynamic SeMo ≥ 5.96 *n* = 201	Dynamic SeMo < 5.96 *n* = 917	OR (95%CI)	*p*
Sex				0.291
Male, *n* (%)	137 (68.2)	589 (64.2)	1.19 (0.86–1.65)	
Female, *n* (%)	64 (31.8)	328 (35.8)	0.84 (0.61–1.16)	
Age	58.4 ± 20.2	58.5 ± 19.2	-	0.978
SeMo (Initial)	13.1 ± 5.9	19.0 ± 10.8	-	<0.001
Neutrophil (count/uL)	8164.4 ± 5336.7	9855.5 ± 4946.4	-	<0.001
Monocyte (count/uL)	680.1 ± 514.8	591.1 ± 344.2	-	0.020
SeMo (Second)	34.3 ± 33.8	12.5 ± 5.0	-	<0.001
Neutrophil (count/uL)	10,043.4 ± 3940.9	7537.1 ± 3341.4	-	<0.001
Monocyte (count/uL)	398.3 ± 235.4	653.1 ± 327.7	-	<0.001
Comorbidities				
DM, *n* (%)	40 (19.9)	193 (21.0)	0.93 (0.64–1.36)	0.717
HTN, *n* (%)	79 (39.3)	346 (37.7)	1.07 (0.78–1.46)	0.678
CAD, *n* (%)	20 (10.0)	70 (7.6)	1.34 (0.79–2.25)	0.274
CHF, *n* (%)	2 (1.0)	9 (1.0)	1.01 (0.22–4.73)	0.986
CVA, *n* (%)	15 (7.5)	48 (5.2)	1.46 (0.80–2.66)	0.215
ESRD, *n* (%)	4 (2.0)	30 (3.3)	0.60 (0.21–1.72)	0.338
GCS, median (IQR)	12 (6–15)	13 (7–15)	-	0.014
3–8, *n* (%)	77 (38.3)	269 (29.3)	1.50 (1.09–2.06)	0.013
9–12, *n* (%)	28 (13.9)	142 (15.5)	0.88 (0.57–1.37)	0.578
13–15, *n* (%)	96 (47.8)	506 (55.2)	0.74 (0.55–1.01)	0.056
ISS, median (IQR)	20 (16–27)	20 (16–25)	-	0.216
1–15, *n* (%)	16 (8.0)	106 (11.6)	0.66 (0.38–1.15)	0.138
16–24, *n* (%)	99 (49.3)	480 (52.3)	0.88 (0.65–1.20)	0.427
≥25, *n* (%)	86 (42.8)	331 (36.1)	1.32 (0.97–1.81)	0.076
Mortality, *n* (%)	43 (21.4)	78 (8.5)	2.43 (1.94–4.15)	<0.001
AOR of mortality ^†^	-	-	2.98 (1.95–4.56)	<0.001
LOS in hospital (days)	23.6 ± 18.2	19.7 ± 15.9	-	0.005

AOR = Adjusted odd ratio; CAD = coronary artery disease; CHF = congestive heart failure; CI = confidence interval; CVA = cerebral vascular accident; DM = diabetes mellitus; ESRD = end-stage renal disease; GCS = Glasgow Coma Scale; HTN = hypertension; IQR = interquartile range; ISS = injury severity score; LOS = length of stay; OR = odds ratio, SeMo = Neutrophil to Monocyte ratio. ^†^, AOR controlled by age, CAD, CVA, ESRD, GCS, and ISS.

**Table 3:** Comparison of patients with dynamic SeMo greater than or equal to 0 to those with a ratio less than 0.

Variables	Dynamic SeMo ≥ 0.00 *n* = 412	Dynamic SeMo < 0.00 *n* = 706	OR (95%CI)	*p*
Sex				0.645
Male, *n* (%)	264 (64.1)	462 (65.4)	0.94 (0.73–1.22)	
Female, *n* (%)	148 (35.9)	244 (34.6)	1.06 (0.82–1.37)	
Age	58.3 ± 19.5	58.6 ± 19.3	-	0.806
SeMo (Initial)	12.5 ± 5.3	21.1 ± 11.2	-	<0.001
Neutrophil (count)	8172.9 ± 5149.0	10,355.9 ± 4829.4	-	<0.001
Monocyte (count)	701.5 ± 477.5	552.0 ± 299.7	-	<0.001
SeMo (Second)	24.3 ± 25.8	11.8 ± 4.9	-	<0.001
Neutrophil (count)	9223.5 ± 3720.8	7266.5 ± 3301.1	-	<0.001
Monocyte (count)	507.8 ± 289.6	665.3 ± 335.3	-	<0.001
Comorbidities				
DM, *n* (%)	83 (20.1)	150 (21.2)	0.94 (0.69–1.26)	0.662
HTN, *n* (%)	159 (38.6)	266 (37.7)	1.04 (0.81–1.34)	0.761
CAD, *n* (%)	35 (8.5)	55 (7.8)	1.10 (0.71–1.71)	0.676
CHF, *n* (%)	5 (1.2)	6 (0.8)	1.43 (0.44–4.73)	0.552
CVA, *n* (%)	25 (6.1)	38 (5.4)	1.14 (0.66–1.91)	0.632
ESRD, *n* (%)	9 (2.2)	25 (3.5)	0.61 (0.28–1.32)	0.203
GCS, median (IQR)	12 (7–15)	13 (8–15)	-	0.024
3–8, *n* (%)	144 (35.0)	202 (28.6)	1.34 (1.03–1.74)	0.027
9–12, *n* (%)	63 (15.3)	107 (15.2)	1.01 (0.72–1.42)	0.951
13–15, *n* (%)	205 (49.8)	397 (56.2)	0.77 (0.60–0.98)	0.036
ISS, median (IQR)	20 (16–26)	20 (16–25)	-	0.507
1–15, *n* (%)	46 (11.2)	76 (10.8)	1.04 (0.71–1.54)	0.836
16–24, *n* (%)	202 (49.0)	377 (53.4)	0.84 (0.66–1.07)	0.158
≥25, *n* (%)	164 (39.8)	253 (35.8)	1.18 (0.92–1.52)	0.185
Mortality, *n* (%)	63 (15.3)	58 (8.2)	2.02 (1.38–2.95)	<0.001
AOR of mortality ^†^	-	-	2.01 (1.30–3.01)	<0.001
LOS in hospital (days)	22.1 ± 17.2	19.5 ± 15.8	-	0.011

AOR = Adjusted odd ratio; CAD = coronary artery disease; CHF = congestive heart failure; CI = confidence interval; CVA = cerebral vascular accident; DM = diabetes mellitus; ESRD = end-stage renal disease; GCS = Glasgow Coma Scale; HTN = hypertension; IQR = interquartile range; ISS = injury severity score; LOS = length of stay; OR = odds ratio, SeMo = Neutrophil to Monocyte ratio. ^†^, AOR controlled by age, CAD, CVA, ESRD, GCS, and ISS.

**Table 4:** Univariate and multivariate analysis of the risk factors for mortality.

Variables	Univariate Analysis			Multivariate Analysis		
	OR	95%CI	*p*	OR	95%CI	*p*
Age	1.02	(1.01–1.03)	<0.001	1.03	(1.02–1.04)	<0.001
CVA	2.37	(1.23–4.56)	0.010	2.03	(0.98–4.22)	0.057
CAD	2.25	(1.31–3.86)	0.003	1.51	(0.81–2.81)	0.192
ESRD	4.93	(2.43–10.02)	<0.001	5.11	(2.32–11.23)	<0.001
GCS	0.86	(0.83–0.89)	<0.001	0.87	(0.83–0.90)	<0.001
ISS	1.06	(1.04–1.08)	<0.001	1.06	(1.04–1.08)	<0.001
Dynamic SeMo	1.01	(1.00–1.02)	0.039	1.01	(1.01–1.03)	0.031

CAD = coronary artery disease; CI = confidence interval; CVA = cerebral vascular accident; ESRD = end-stage renal disease; GCS = Glasgow Coma Scale; ISS = injury severity score; OR = odds ratio.

## Data Availability

The original contributions presented in the study are included in the article, further inquiries can be directed to the corresponding author.
